# The Role of Cancer Stem Cells in Radiation Resistance

**DOI:** 10.3389/fonc.2020.00164

**Published:** 2020-02-20

**Authors:** Christoph Reinhold Arnold, Julian Mangesius, Ira-Ida Skvortsova, Ute Ganswindt

**Affiliations:** ^1^Department of Therapeutic Radiology and Oncology, Medical University of Innsbruck, Innsbruck, Austria; ^2^EXTRO-Lab, Tyrolean Cancer Research Institute, Innsbruck, Austria

**Keywords:** cancer stem cells, radiation resistance, radiation therapy, DNA damage repair, reactive oxygen species, stem cell niche, quiescence

## Abstract

Cancer stem cells (CSC) are a distinct subpopulation within a tumor. They are able to self-renew and differentiate and possess a high capability to repair DNA damage, exhibit low levels of reactive oxygen species (ROS), and proliferate slowly. These features render CSC resistant to various therapies, including radiation therapy (RT). Eradication of all CSC is a requirement for an effective antineoplastic treatment and is therefore of utmost importance for the patient. This makes CSC the prime targets for any therapeutic approach. Albeit clinical data is still scarce, experimental data and first clinical trials give hope that CSC-targeted treatment has the potential to improve antineoplastic therapies, especially for tumors that are known to be treatment resistant, such as glioblastoma. In this review, we will discuss CSC in the context of RT, describe known mechanisms of resistance, examine the possibilities of CSC as biomarkers, and discuss possible new treatment approaches.

## Introduction

Radiation therapy (RT) is one of the mainstays of cancer treatment. Roughly one half to two-third of all oncologic patients receive some form of RT in the course of their disease (1–5), either in a curative setting for primary treatment with or without other treatment modalities (i.e., surgery or chemotherapy, CHT) or in a palliative setting for the irradiation of symptomatic metastases. Importantly, the number of patients requiring RT is expected to increase in the foreseeable future (6). In short, RT exerts its effect by inducing DNA damage, either directly or indirectly via the production of water-derived radicals and reactive oxygen species (ROS) (7–9), which then interact with macromolecules including DNA, lipids and proteins. As a consequence, DNA damage response (DDR) is initiated and leads to the activation of the DNA damage repair machinery as well as the induction of checkpoint kinase pathways, which delay cell cycle progression in order to facilitate DNA repair (10–13). In case the DNA is damaged beyond repair, DDR signaling induces apoptosis, senescence or mitotic catastrophe, all of which imply the loss of reproductive capacity of a cell (14–16). Consequently, if successful, RT hinders cancer cells from further proliferation. In theory, every (cancerous) cell can be killed with RT given a high enough dose. However, the surrounding healthy tissue limits the applicable dose (17). RT is usually a balancing act between giving enough dose to achieve local tumor control and only as much dose as the surrounding tissue can tolerate. Despite a very high local tumor control rate, a non-negligible rate of therapy failure still constitutes one of the major limitations in radiation oncology (18, 19). Insufficient response to irradiation (i.e., radiation resistance) contributes to residual cancer mass, which is the key driver of locoregional or distant recurrence, both of which are negatively influencing the patient's prognosis as local recurrence often is associated with metastatic spread, which is almost always fatal.

In recent years evidence has accumulated showing that multiple genetically diverse clones co-exist within various kinds of tumors (20–24). Not all cells within a tumor are equally sensitive to RT. Understanding the diverse radiosensitivity of different tumor cell subpopulations is very important. It challenges the common practice of employing macroscopic bulk tumor responses (as measured with medical imaging) as the primary endpoint for determining the effectiveness of an anti-neoplastic treatment. While this is certainly a very practical approach, it is only then true if the bulk tumor response represents the response of all cells within the tumor, including the most resistant subpopulation within the tumor. This is most likely not the case for most tumors because not all subpopulations are equally affected by the treatment. The stem cell model of cancer development may explain genetic, functional, and phenotypical differences, such as increased therapy resistance, within a tumor, even within the same tumor clone. Cancer stem cells (CSC), albeit difficult to identify, are believed to contribute to resistance to various oncological therapies, including RT (25–34), making them a primary target for anti-cancer therapy. Hence, a proper understanding of differential sensitivity of cancer cells, especially CSC, to irradiation is vital in order to develop new or improve existing anti-cancer therapies. Most research on CSC has been done in breast cancer and glioma (35, 36). However, as CSC differ between entities results cannot be transferred to other tumors, at least not without caution.

## Cancer Stem Cells

Today, there are basically two largely accepted models for the origin of cancer: the standard (hierarchical) CSC model and the clonal evolution model. In the latter, genetic mutations accumulate with time and theoretically any cell can have tumorigenic potential (37). The CSC model describes a hierarchical organization of tumors with tumorigenic CSC at the apex which divide asymmetrically to form new CSC as well as differentiated non-tumorigenic progenies (34). Adding to the complexity of the CSC theory, is the fact that differentiation may be bidirectional. In this way, differentiated non-tumorigenic tumor cells may, instructed by niche signals, re-differentiate into CSC to replace lost stem cells. Even though data supporting the CSC hypothesis with its hierarchical organization of tumors is more solid, it is feasible that both, the CSC hypothesis and the clonal evolution model are not necessarily mutually exclusive.

The generation of CSC is not conclusively clarified, and several hypotheses exist (38). CSC may originate from normal stem cells, where random mutations during DNA replication may lead to them becoming malignant (39). Additionally, aberrant stromal signaling and pro-inflammatory conditions can lead to the malignant degeneration of normal stem cell (40). Alternatively, as stated earlier, CSC can be derived from differentiated cells. This can occur via genomic instability of tumor cells, horizontal genetic transfer, or microenvironmental signals. Genetic instability describes the acquisition of additional genetic mutations that provide any given differentiated tumor cell with stem cell traits so that it becomes a CSC (41). It is, however, unclear, whether stem cell traits shift from one cell to another in a stochastic manner during tumor evolution. In horizontal gene transfer, a normal stem cell may phagocyte fragmented DNA from tumor cells leading to their reprogramming and CSC formation (42). Microenvironmental signals include pro-inflammatory cytokines such as interleukin-6 (IL-6), which has been shown to facilitate dedifferentiation of non-CSC into CSC, or nuclear factor-kB (NF-kB), which maintains CSC numbers (43).

The frequency of CSC within a tumor is difficult to estimate and largely depends on the type of malignancy. In solid tumors, reported CSC rates are in the range from below 1% of all tumor cells to more than 80% (44–48). Similarly, the frequency of CSC in hematologic malignancies also displays a broad spectrum and ranges from <1% in acute myeloid leukemia (AML) up to over 80% in acute lymphoblastic leukemia (ALL) (49, 50). It has been shown in glioblastoma that CSC seem to reside predominantly in niches that are hypoxic, low in nutrients and have a low pH (51).

CSC are a distinct subpopulation within the heterogenous tumor mass and share several properties with normal stem cells (SC), the most important being the ability to self-renew (i.e., the potential of unlimited cell division) and the ability to give rise to more differentiated, mature cancer cells (34). Like in healthy tissue, stem cells initiate, promote, and maintain tumor development and growth (and re-growth after treatment) (52–55). It has been shown in glioma and breast cancer that the number of CSC in a tumor at the time of treatment is inversely correlated with clinical outcome (56, 57). Furthermore, repopulation of CSC after fractionated RT is one of the most important factors that determine local tumor control (58, 59). Therefore, inactivation of all CSC within a tumor is the prerequisite for a curative cancer treatment (60).

One major challenge regarding CSC is their correct identification as there is not one specific CSC marker, not least because of the high intra- and inter-tumor heterogeneity as well as tumor plasticity and the associated changes in genotype and phenotype. However, there are some cell surface marker that seem robust enough to use them as indicators for CSC. Two of these biomarkers are CD44 (found on CSC in cancers of the colon, esophagus, stomach, pancreas, breast, brain, lung, ovaries, prostate, liver, and the head and neck region) and CD133 (found in cancers of the colon, esophagus, stomach, pancreas, brain, lung, ovaries, prostate, liver, skin, and the head and neck region) (38, 61). Naturally, there are more surface molecules and usually combinations of these markers are used to identify and isolate CSC depending on the type of tumor that is investigated. It is important to emphasize that CSC differ between tumor entities, both phenotypically and functionally, and results from one type of cancer should not be translated to other types.

## Radiocurability and Radiation Therapy Resistance

Following RT-induced DNA damage the balance of pro- and anti-apoptotic pathways skews toward cell death induction. However, in CSC pro-survival pathways seem to be more pronounced and protect these cells from cell death, rendering CSC radiation resistant (27). Radiation resistance of CSC may either be primary, i.e., due to the constitutive upregulation of certain molecules and pathways (see below). Alternatively, radiation resistance of CSC may be acquired. Following RT, as is the case with any other antineoplastic therapy, intratumoral heterogeneity can theoretically promote clonal evolution through Darwinian selection and lead to the development of adaptive responses with the result of more resistant, aggressive, and invasive tumors (62). CSC clones with genomic alterations that protect them against RT are selected for and continue to sustain the tumor (63). Indeed, it is known that RT preferentially kills non-CSC, thereby enriching the tumor for CSC (64). In addition, RT has been shown to induce the reprogramming of non-CSC in breast cancer as well as squamous cell carcinoma of the head and neck (HNSCC) leads to the acquisition of functional CSC traits in order to compensate for cell loss in the stem cell compartment in response to cellular injury as is the case after RT (65, 66). Finally, RT leads to the recruitment of CSC in breast cancer from a quiescent state into the cell cycle (67, 68). In this way, RT contributes to the acquired or adaptive radioresistance via selective repopulation from the surviving CSC.

The number of CSC within a tumor predicts the radiation dose needed to eradicate the tumor. Therefore, in tumors with a higher proportion of CSC a given dose of irradiation leads to a lower probability of local control as compared to tumors with fewer CSC (69–71). From a clinical point of view, this implies a dose-volume dependency, as radiocurability of tumors inversely correlates with tumor volume (72) and with intrinsic radiosensitivity *in vitro* (73). Furthermore, the probability of successful irradiation also correlates with the number, density, and intrinsic radiosensitivity of CSC (60, 71) and the absolute number of CSC increases with tumor volume (70–72, 74). Importantly, survival of one single CSC after RT can lead to tumor relapse. Hence, eradication of the entire CSC population is of utmost importance for the patient. Nonetheless, one must keep in mind that CSC differ between tumor types and there is no general radiation resistance of CSC, as many patients can be cured with current concepts of conventional RT.

## Cellular Factors for CSC Radioresistance

**Figure 1:**
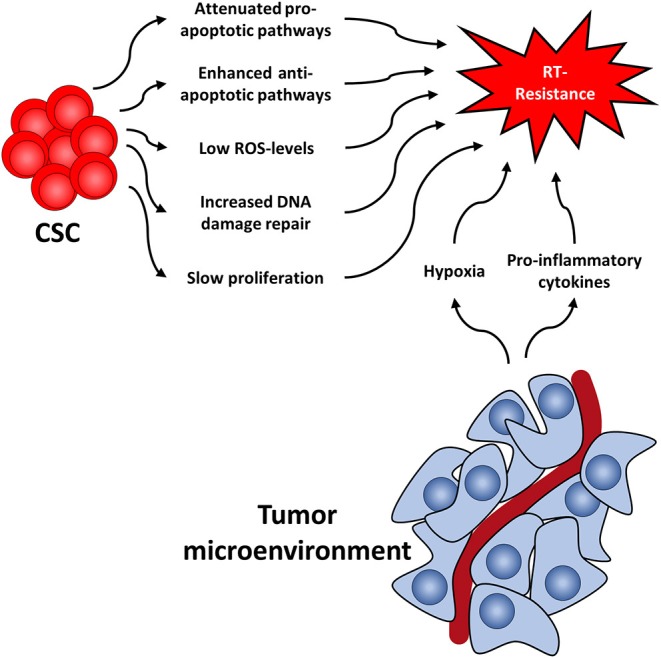
Cancer stem cell-related factors as well as the tumor microenvironment both contribute to radioresistance and reveal new therapeutic approaches.

Several cellular features render CSC radioresistant. In the following, we will discuss the best-studied cellular factors, which include low levels of ROS, increased DNA damage repair capacity, or quiescence (Figure 1). These are common characteristics of healthy SC and CSC alike, presumably to protect their DNA from stress-induced damages.

ROS are involved in various physiological processes, such as proliferation, differentiation, metabolism, apoptosis, angiogenesis, wound healing, or motility (75, 76). Intracellular ROS levels are tightly and continuously regulated by ROS scavengers, which include superoxide dismutase, superoxide reductase, catalase, glutathione peroxidase, glutathione reductase, or apurinic/apyrimidinic endonuclease/redox effector factor (Ape1/Ref-1, also known as APEX1) (77). There is a multitude of publications showing that ROS scavengers are upregulated and highly efficient in CSC of various tumors (78–83) leading to low levels of ROS and protecting CSC from RT-induced cell death, as ROS is essential for the effect of RT (84). This protective effect of upregulated ROS scavengers even outweighs the effect of oxygen, a known potent radiosensitizer (85). Along this line, it has been shown that CSC produce less ROS upon radiation compared to non-CSC (86).

Secondly, DNA damage repair capacity following RT, especially regarding DNA double strand breaks (DSB), has been shown to be higher in CSC as compared to their non-tumorigenic counterparts (25, 87–90). This has been shown in CSC of several tumors, including glioma, nasopharyngeal carcinoma, prostate, lung, and breast cancer, and is mainly attributed to the activation of checkpoint-pathways in response to RT (90–98). The resulting delayed cell cycle progression allows for repair of the DNA damage. Interestingly, CSC have been shown to repair DNA damage preferably via homologous recombination (HR) instead of non-homologous end-joining (NHEJ), the latter being less accurate and more error-prone than HR (25, 99–101). A comprehensive review on DNA damage repair in CSC has recently been published (102).

Third, it has been shown in various studies that CSC proliferate more slowly than further differentiated cancer cells (78, 103, 104). This is of importance as RT is known to be more effective in killing rapidly proliferating tumor cells as compared to slowly dividing (i.e., quiescent or dormant) cells and quiescence is associated with relative radiation resistance (105, 106). This way, non-proliferating cells survive therapeutic irradiations and remain quiescent for a various amount of time, which can range from weeks (78, 104) to even decades (107, 108). Once they continue to proliferate, these cells can cause a recurrence.

## Tumor Microenvironment

Similar to healthy stem cells, CSC reside in specific niches that provide microenvironmental factors such as autocrine signaling and signals coming from stromal fibroblasts (cancer associated fibroblasts, CAF), immune cells, endothelial cells, and extracellular matrix components (109, 110). The exact composition of the niche is not well-defined for most tumors, as are the exact supporting signals. It is known, however, that the niche supplies CSC with oxygen and nutrients, supports stem cell functions, protects from insults such as radiation, and regulates responsiveness to a therapy (111). For instance, in breast cancer it has been shown that deregulation of the stem cell niche by increased expression of bone morphogenetic protein 2 (BMP2) can initiated and promote malignant stem cell transformation (112). Furthermore, at least in glioblastoma tumor samples, there are different types of niches, such as hypoxic peri-arteriolar niches, peri-vascular niches, peri-hypoxic niches, peri-immune niches, and extracellular niches (113). Whether other types of cancer have different types of niches, and what therapeutic implications this finding might have, still needs to be elucidated.

Like the tumor itself, the tumor microenvironment also responds to RT (114) (Figure 1). For instance, it has been shown that CAFs acquire a pro-malignant phenotype after RT of in colorectal cancer samples (115–117). Furthermore, CAFs induce autophagy following irradiation of lung cancer and melanoma cell lines leading to enhanced cancer cell recovery and tumor re-growth (118).

Additionally, RT induces pro-inflammatory cytokines in the tumor microenvironment (119, 120), including platelet-derived growth factor (PDGF), interleukin 1β (IL1β), tumor necrosis factor α (TNFα), transforming growth factor β (TGFβ), C-X-C motif chemokine 12 (CXCL12), and matrix metalloproteinases (MMP), and interleukin-6 (IL6). This leads to the upregulation of ROS scavengers in CSC (7) and activation of downstream STAT3 signaling, a cascade known to promote self-renewal in embryonic stem cells neu (121). Furthermore, this promotes survival of tumor cells, facilitates tumor regrowth and leads to the development of highly invasive CSC phenotypes (122, 123).

Another mechanism by which the niche protects CSC is hypoxia. Oxygen is a potent radiosensitizer and is needed for radiation-induced ROS production and in further consequence for cell death. Lack of oxygen is known to increase radiation resistance (124–126) and has been associated with early relapse after RT. Consequently, increasing tumor oxygenation improves the response to RT (127–129). In addition to the absolute lack of oxygen and the resulting low ROS levels, CSC in hypoxic niches upregulate ROS scanvengers (7, 130, 131), thereby further lowering ROS levels compared to normoxic CSC. This in turn leads to the activation of the hypoxia-inducible factor (HIF) signaling route (132–134). Interestingly, HIF1α and the respective regulated cytokines have also been shown to be increased following RT (135). HIF are important master regulators of transcription of hypoxia response elements, which activates pro-survival pathways such as the Notch, wingless and INT-1 (WNT) and Hedgehog pathway (136–138). These pathways have been shown to be important for CSC maintenance and can lead to radioresistance and accelerated repopulation of CSC during or after treatment, as has been shown in glioma, breast cancer, and prostate cancer (97, 139–142).

## CSC as Biomarker

There is accumulating evidence that CSC could be used as biomarkers to predict treatment response and estimate the likelihood of tumor relapse in cancer patients. It has been shown in various tumors, including urothelial cancer (143–145), gastric cancer (146–150), pancreatic cancer (151, 152), HNSCC (153–155), glioma (156–159), thyroid carcinoma (160, 161), hepatocellular carcinoma (162, 163), breast cancer (164), and lung cancer (165). However, it has been shown in ovarian cancer that the prognostic value of CD44 may depend on its isoform, with the transmembrane form indicating a better prognosis, while the presence of the soluble extracellular domain was associated with a worse prognosis (166). In a recent meta-analysis, overall CD44 expression in ovarian cancer was associated with a high TNM stage and a poor 5 year overall survival (167). Along this line, low expression of CD44 was shown to be an independent factor of poor prognosis in ovarian mucinous carcinoma (168). Interestingly, in breast cancer, it has been shown that CD44 was associated with longer disease-free-survival (DFS) in estrogen-receptor (ER) positive women, while CD44 positive tumors were associated with poor outcome in ER-negative patients (169). In a recent meta-analysis, Han and colleagues tried to generalize the prognostic significance of CD44 and its variant isoforms in advanced cancer patients. In this analysis of 15 articles with more than 1,200 patients, CD44 was slightly linked to a worse overall survival, but there was no correlation between CD44 expression and DFS, recurrence-free survival (RFS), or progression-free survival (PFS) (170).

Two studies from the German Cancer Consortium Radiation Oncology Group (DKTK-ROG) have shown that CSC marker expression is a potential biomarker for favorable prognosis in patients with locally advanced HNSCC, both after primary chemoradiotherapy (171) as well as following post-operative chemoradiotherapy (172). In a recent validation study from the same group, the addition of CD44 could further improve the prognostic performance of models using tumor volume, p16 status, and N stage (173).

Activity of the 26S proteasome, a protease complex with regulatory functions in cell cycle, DNA repair, and cell survival, is another CSC marker (131, 174–177). In this regard, low 26S proteasome activity correlated with high self-renewal capacity and high tumorigenicity in HNSCC cell lines (178). A high 26S proteasome activity correlated with a longer survival and higher local control rates in patients who underwent chemoradiotherapy for HNSCC (95).

Another potential biomarker, especially for glioma, might be the stem cell marker CD133. In a recent meta-analysis including 21 articles with more than 1,550 patients, CD133 expression correlated with higher grade of gliomas and worse prognosis in glioma patients (179). Interestingly, in a recent *in vitro* analysis in glioma cell lines, CD133 expression could be downregulated by vincristine, a common chemotherapeutic drug (180). In a study by Wu and colleagues, CD133 promotor methylation was a significant prognostic factor for adverse PFS and overall survival, while there was no correlation between CD133 protein expression and survival (181). Additionally, there are other publications that showed no association of CD133 protein expression with survival (182, 183).

## Novel Treatment Approaches

Increased understanding of CSC has led to new ideas for improving cancer therapy. It certainly seems feasible to combine conventional anti-neoplastic therapy (e.g., RT or CHT) to target the tumor bulk with CSC specific treatment in order to improve outcome as compared to monotherapies (184, 185). Inactivation of even only limited numbers of CSC might significantly improve local tumor control probability (70). However, the assumed plasticity of the CSC and non-CSC compartments, especially the possible shift of stem cell traits, increases the complexity of treatment responses of tumors. In an ideal situation where the CSC population is strictly static, drugs that specifically target CSC would lead to a massively improved treatment outcome, possibly even without the need of additional treatment modalities (e.g., RT or CHT). However, CSC plasticity render a CSC-targeting monotherapy virtually impossible, since after treatment, non-CSC may gain CSC traits and repopulate the tumor. Additionally, regarding the current CSC marker and their uncertainty in robustly identifying CSC, and to sufficiently distinguish them from healthy SC, a strictly CSC-based therapy seems to be still a long way off.

One conceivable possibility to eliminate CSC with RT more efficiently is to increase the applied dose. This is usually not feasible for the whole tumor due to dose constraints of the surrounding healthy tissue. Therefore, visualization of CSC could allow for larger doses of radiation in CSC rich regions while still respecting the dose constraints. Indeed, there are first studies in mice, where CD133+ glioblastoma cells could be detected non-invasively by PET and near-infrared fluorescence molecular tomography using antibody-labeled tracer (186). Subsequently, the same group showed that near-infrared photoimmunotherapy using phototoxic antibody conjugates was efficient not only in rendering CD133+ glioblastoma cells visible but also in inducing cell death (187).

Another means by which RT can be utilized to eliminate CSC more efficiently is the use of types of irradiation other than commonly used photon beams (188, 189). Particle beams of protons and carbon ions are being increasingly used due to their advantageous depth-dose curve and their higher cell-killing efficiency compared with photons (190). Preclinical studies deliver promising results when using proton irradiation. For instance, in CSC-like cells from two human NSCLC cell lines, irradiation with protons was more efficient than photon treatment in reducing cell viability. clonogenic survival, cell migration, and invasiveness, while increasing apoptosis and ROS levels (191). Furthermore, proton irradiation has been shown to be more cytotoxic, induce higher and longer cell cycle arrest, reduce cell adhesion and migration ability, and reduce the overall population of CSC in NSCLC cell lines compared to photon irradiation (192). Finally, in glioma stem cells from glioblastoma patients, similar results have been achieved (193). In this study, particle irradiation with protons and carbon ions has been shown to be more effective in cell killing compared to photons, likely because of the different quality of the induced DNA damage. Indeed, compared to photons, proton beam irradiation has been shown to increase ROS levels, induce more single and double strand DNA breaks, less DNA damage repair (as measured by H2AX phosphorylation), and decreased cell cycle recovery which led to increased apoptosis (194). Interestingly, primary human glioma stem cells that were resistant to photon treatment could be rendered sensitive with carbon ion irradiation via impaired capacity to repair carbon ion induced DNA double strand breaks (195). Importantly, this study also showed an individual heterogeneity in the amount and radiosensitivity of glioma stem cells from different patients, further complicating a one size fits all treatment. In the recent years, immune checkpoint inhibitors have greatly improved treatment outcomes for many cancers. In this regard, it has been shown that proton irradiation increased sensitivity of CSC from different cell lines, such as breast or prostate cancer, to cytotoxic T-cell killing (196). These findings offer a rationale for the combined use of proton irradiation with immunotherapy. Another important aspect of particle irradiation is the reduced dependence from tissue oxygenation. While photon irradiation is always strongly affected by the presence of oxygen in the induction and maintenance of DNA damage, high LET particle beams can be much less hindered by hypoxic conditions, which are often found in solid tumors. For example, Tinganelli et al. (197) showed the survival of mammalian cells exposed to different types of particle radiation in various oxygen concentrations, leading to a hypoxia-adapted irradiation plan. Hence, it seems feasible and promising to use particle irradiation in order to counteract tumor hypoxia. Taken together, these results suggest a potential advantage of particle beam irradiations in CSC eradication, eventually in combination with conventional photon irradiation: photon beam irradiation to the whole tumor and a boost of particle beams to hypoxic areas within the tumor. Alternatively, one can ideally use a properly optimized plan of carbon or oxygen beam, or, considering the potential of the most advanced particle centers, a multiple-ion irradiation (198). Another new strategy in combating glioblastoma is the interference with metabolic pathways. It has been shown that dichloroacetate (DCA), an orphan drug, has been shown to switch the metabolism in freshly isolated glioma stem cells from mitochondrial oxidative phosphorylation to cytoplasmic glycolysis, which in turn increased mitochondrial ROS and induced apoptosis (199). In the same study, glioblastoma patients treated with the standard of care (i.e., radiation therapy and temozolomide) received oral DCA for up to 15 months. The drug was well-tolerated, and some patients showed prolonged radiologic stabilization and decelerated tumor progression. Additionally, DCA, in combination with PENAO [4-(N-(S-penicillaminylacetyl)amino) phenylarsonous acid] has been shown to increase radiosensitivity of glioma cells by inducing a cell cycle arrest, elevating ROS production, depolarizing mitochondrial membrane potential, increasing DNA damage, and inducing apoptosis (200).

Early experiments with CSC-directed antibodies have shown promising results. Gurtner et al. (201) used antibodies directed against CD44 that were loaded with highly cytotoxic drugs. In this experimental setting, these antibodies, combined with irradiation, led to an improved local tumor control. Considering the high expression of ROS scavengers in CSC, it may be reasonable to target these ROS scavengers. Pharmacological depletion of ROS scavengers (e.g., by treatment with buthionine sulfoximine (BSO), has been shown to reduce radiation resistance as well as clonogenic properties of CSC in breast cancer and HNSCC (202, 203). Additionally, the use of antioxidants during RT, which reduce ROS levels, might prove nonsensical, as high ROS levels are needed, especially in CSC, in order to facilitate RT-induced cell death.

Checkpoint pathways can be inhibited pharmacologically to prevent delayed cell cycle progression and hamper DNA damage repair in CSC as has been shown in glioma and prostate cancer (98, 204). Additionally, direct inhibition of DNA damage repair signaling has been shown to reduce radiation resistance in breast cancer CSC (92). Chemical inhibition of Notch signaling, a developmental pathway that is known to be essential for tissue homeostasis (205) has been shown to increase radiosensitivity in glioma CSC (206). However, most clinical studies regarding these signaling pathways focus on chemotherapy (207). There is preclinical data in mice with glioblastoma multiforme that were treated with RT and temozolomide combined with a Notch inhibitor. In this study, Notch inhibition had an anti-glioma stem cell effect which provided a survival benefit (208). Regarding clinical data, there is an ongoing trial testing a Notch inhibitor combined with whole-brain RT or stereotactic cranial RT [NCT01217411], but so far there are no results of this study available.

Overcoming tumor hypoxia is another method to improve cancer therapy. Nitroimidazole derivates can mimic the effect of oxygen and can produce reactive species in hypoxic cells. There have already been clinical trials with a clinically relevant sensitization and low toxicity (209, 210). Nitric oxide (NO) is also able to mimic oxygen and thereby to increase radiosensitivity in hypoxic tissue. In a first clinical trial, the NO donor glyceryl-trinitrate (GTN) has been shown to reduce hypoxia-induced progression of prostate cancer (211). Taken together, targeting the hypoxic niche of CSC might eventually improve treatment outcome following RT. An important task in this regard will be the correct identification of CSC for a given tumor, since CSC can differ between tumors both functionally/metabolically as well as phenotypically. Additionally, CSC share many properties and surface molecules with normal stem cells, making a clear distinction difficult and increasing the risk of unwanted side effects. Finally, it seems conceivable that drugs that interfere with spontaneous as well as RT-induced reprogramming of non-CSC into CSC could also be of value for cancer treatment. However, it needs to be investigated if these mechanisms are the same in normal steam cells and CSC before such drugs can be developed.

Another interesting approach to amplify the effect of radiation therapy, particularly on CSC, is the use of nanostructures that, after being endocytosed by cancer cells and following irradiation, release secondary electrons and large amounts of ROS (212). This could be especially effective in CSC, where ROS levels are generally lower. In this study, a significant tumor growth suppression and overall improvement in survival rate has been demonstrated in an *in vitro* and *in vivo* model of triple negative breast cancer.

## Conclusion

There is growing evidence of a radiation resistance tumor subpopulation with increased DNA damage repair, increased survival signaling, and decreased ROS, that is furthermore protected by its environment. With refined understanding of these cells and their role in development and progression of cancer come new possibilities to improve cancer therapy. Targeting CSC, based on phenotype or function, seems promising. Nonetheless, we are just at the beginning and clinical data is still scarce. Major issues concern their correct identification and reliable distinction from healthy cells and the plasticity of the CSC department. It will be exciting to see which position CSC-specific therapies will occupy within the row of current anti-neoplastic therapies.

## Author Contributions

CA and JM performed the literature research. CA wrote the manuscript. I-IS and UG supervised the project.

### Conflict of Interest

The authors declare that the research was conducted in the absence of any commercial or financial relationships that could be construed as a potential conflict of interest.
